# Feature Selection Techniques for a Machine Learning Model to Detect Autonomic Dysreflexia

**DOI:** 10.3389/fninf.2022.901428

**Published:** 2022-08-10

**Authors:** Shruthi Suresh, David T. Newton, Thomas H. Everett, Guang Lin, Bradley S. Duerstock

**Affiliations:** ^1^Weldon School of Biomedical Engineering, Purdue University, West Lafayette, IN, United States; ^2^Department of Statistics, Purdue University, West Lafayette, IN, United States; ^3^Krannert Cardiovascular Research Center, Indiana University School of Medicine, Indianapolis, IN, United States; ^4^School of Mechanical Engineering, Purdue University, West Lafayette, IN, United States; ^5^Department of Mathematics, Purdue University, West Lafayette, IN, United States; ^6^School of Industrial Engineering, Purdue University, West Lafayette, IN, United States

**Keywords:** spinal cord injuries, machine learning, feature selection, electrocardiography, healthcare

## Abstract

Feature selection plays a crucial role in the development of machine learning algorithms. Understanding the impact of the features on a model, and their physiological relevance can improve the performance. This is particularly helpful in the healthcare domain wherein disease states need to be identified with relatively small quantities of data. Autonomic Dysreflexia (AD) is one such example, wherein mismanagement of this neurological condition could lead to severe consequences for individuals with spinal cord injuries. We explore different methods of feature selection needed to improve the performance of a machine learning model in the detection of the onset of AD. We present different techniques used as well as the ideal metrics using a dataset of thirty-six features extracted from electrocardiograms, skin nerve activity, blood pressure and temperature. The best performing algorithm was a 5-layer neural network with five relevant features, which resulted in 93.4% accuracy in the detection of AD. The techniques in this paper can be applied to a myriad of healthcare datasets allowing forays into deeper exploration and improved machine learning model development. Through critical feature selection, it is possible to design better machine learning algorithms for detection of niche disease states using smaller datasets.

## Introduction

Current healthcare practices revolve around human expert assessments of correlations between symptoms and diagnoses. There is a growing trend in the medical community to use automated or semi-automated systems to monitor the well-being of individuals in their care. Several of these automated systems leverage upon machine learning (ML). ML has been applied to various areas of healthcare and has enormous potential to improve detection of disease for rapid point-of-care treatment (Saria et al., 2010; Kuhn and Johnson, 2013; Alimadadi et al., 2020; Mishra et al., 2020), help clinicians with making diagnostic decisions (decision support system) (Roski et al., 2014; Rumshisky et al., 2016; Esteva et al., 2017), and improve individual management of chronic health conditions.

Machine learning techniques can contribute to finding patterns and trends that contribute to the knowledge about different disease states as well as help diagnose them early (Chen et al., 2017). Supervised ML methods are among some of the most common approaches used in the clinical setting due to the large amount of annotated data which is available (Wiens and Shenoy, 2018). Some applications of ML to healthcare settings include automated arrhythmia analysis tools using physiological data such as electrocardiogram (ECG) or alerts for low oxygen saturation using photoplethysmography (PPG) (Polat and Gunes, 2007; Uçar et al., 2017; Alfaras et al., 2019; Radha et al., 2019). However, despite its strengths, ML cannot identify relationships that are not present in the data; therefore, data veracity is critical to any accurate ML model (Wiens and Shenoy, 2018). Supervised ML methods are comprised of three crucial steps- feature extraction and selection, classifier training, and lastly evaluation (Badillo et al., 2020).

Feature extraction is the process of reducing a set of raw/preprocessed data into a smaller set of features which represent the key qualities of the data. In healthcare data extraction of relevant features is often guided by physiological understanding of the mammalian system (Jen et al., 2012; Su et al., 2012; Jothi and Husain, 2015). Feature selection prevents overfitting of a machine learning model to improve performance and provide faster, more cost-effective models. Through feature selection, the original representation of the features is not altered, and the original semantics are preserved. Additionally, through specific feature selection, we can gain deeper insight into the underlying processes which led to variation in the data. Automated feature selection through deep learning networks have also been explored in healthcare literature (Waring et al., 2020; Wosiak and Kowalski, 2020). Despite their ability to select relevant techniques and features rapidly, they can limit comprehension of the phenomenon being classified. Additionally, they rely heavily on large amounts of data which may not be common in various medical datasets. Once relevant features have been identified from the data, machine learning models can be trained and evaluated. There are a myriad of feature selection techniques and machine learning models which have been used in various biomedical applications.

In this paper, we present the feature selection techniques and supervised machine learning models we explored in the development of a system for the detection of autonomic dysreflexia (AD). AD is a potentially life-threatening disorder which occurs in individuals with spinal cord injuries (SCI) due to often innocuous triggers below the level of injury. Self-management of AD begins with individuals understanding their symptoms and knowing triggers. Very few researchers have explored the detection of AD while it occurs. These studies rely entirely on the pre-determined patterns in blood pressure measured by a telemetry system to detect the onset of AD event induced by a trigger (Rabchevsky et al., 2012; Popok et al., 2016). However, there are few studies known to the authors which explore the use of multimodal systems to detect the onset of AD. Particularly, there are no studies which have explored the use of machine learning algorithms to automate the process of detecting AD during onset using non-symptom-based approaches.

We developed a non-invasive, multi-parametric system to detect AD using the most efficient machine learning methods and feature selection techniques (Suresh and Duerstock, 2020). In this paper, we describe the feature extraction and selection procedures required to develop an efficient machine learning model which can characterize the onset of AD. These feature selection techniques can also be applied in a variety of medical applications which do not have large datasets due to the relatively small population of persons with this condition.

## Materials and Methods

### Dataset Preparation

Sensor data was collected from 19 male Sprague Dawley rats. All animals were between 3 and 5 months of age and weighed 450–600 g prior to spinal cord injury. These rats were given a spinal cord injury at the T2/T3 level and AD was induced through colorectal distension (O’ Mahony et al., 2012) up to 14 days post-SCI. All rats had sensory and motor loss below the level of injury which was verified through pinch tests. The experiments were performed in accordance with the international directions for the protection of animals used for scientific purposes and the protocol was approved by the Purdue University IACUC.

#### Sensors

**FIGURE 1:**
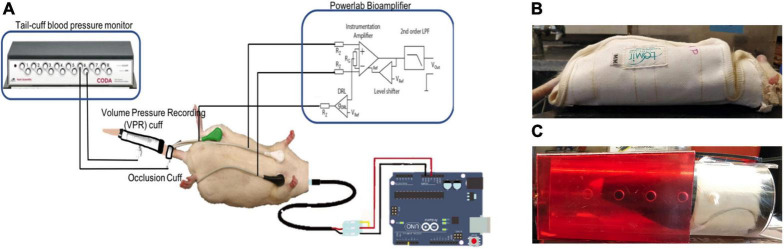
**(A)** Schematic of the sensors. Noninvasive electrodes placed on the ventral skin surface of a rat in Lead I configuration, the Coda^®^ Blood Pressure system with occlusion and VPR cuff and a temperature probe connected to an Arduino^®^. Rats restrained in **(B)** Lomir^®^ “cuddle” jacket and **(C)** Plexiglass tube to restrain the animal during data collection.

Time-series data were collected from wearable ECG, skin nerve activity (skNA), blood pressure (BP), and skin temperature sensors from a restrained animal while it was awake (Figure 1). skNA allows non-invasive measurement of stellate ganglion nerve activity which provides sympathetic innervation to the heart, and has been validated in humans, rat and dog models (Jiang et al., 2015; Everett et al., 2017; Suresh et al., 2019).

Electrocardiogram and skNA were measured through gel-based electrodes placed in a Lead I configuration at the level of the right and left third ribs, with the electrode placed at the right leg serving as a reference electrode. Placement of the electrodes in this location allowed us to observe the cardiovascular activity below the level of injury. Since most AD symptoms are related to the cardiovascular system, the location of the electrodes allow us to investigate the association of nerve activity to cardiovascular impacts during AD. The electrodes were connected to the Power Lab 26T bio-amplifier (AD Instruments, Colorado Springs, CO, United States) and digitized with a sampling rate of 10 kHz and a recording bandwidth of 10 Hz–3 kHz (Jiang et al., 2015).

Blood pressure (BP) was measured through a CODA 6-Channel High Throughput Non-Invasive Blood Pressure system (Kent Scientific, United States) (Daugherty et al., 2009). The Coda system provides measurements of the systolic (SBP), diastolic (DBP) and mean (MAP) blood pressure from the tail of the animal. The BP values were measured two times a minute. The blood pressure system comprises an occlusion cuff placed at the base of the tail and a volume-pressure recording (VPR) cuff which is placed 2 inches from the base of the rat’s tail.

A DS18B20 waterproof digital temperature probe was used to measure skin temperature from the shaved back of the rat directly above the site of injury. The temperature probe is connected to an Arduino and provides up to 12 bits of temperature data from the onboard digital to analog controller (Maxim, 2008). In conjunction with the Dallas temperature control Arduino library, the temperature sensor logs data with a sampling rate of 0.03 Hz (LI Gang, 2005).

Variations in the sampling rate were adjusted post-processing through timestamp matching. A 20 mmHg increase in systolic blood pressure when colorectal distension was induced was used as a gold standard to label the data collected from certain timestamps as either AD or non-AD datapoints.

#### Signal Processing

**FIGURE 2:**
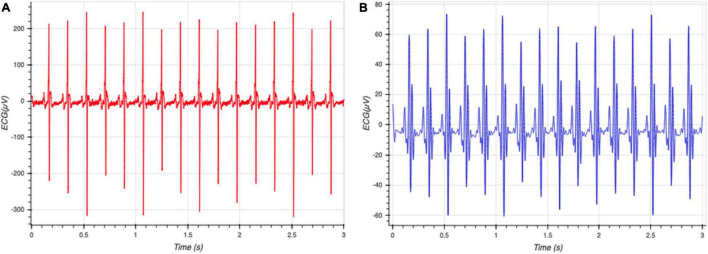
**(A)** Raw ECG data collected from rats **(B)** processed with ECG without high frequency components and prominent R and S segments. This allows clear determination of individual beats of the ECG signal.

**FIGURE 3:**
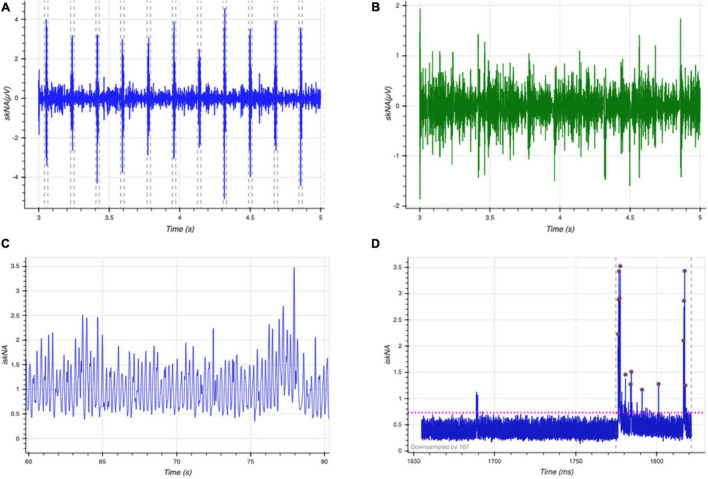
**(A)** Raw skNA signal with QRS interferences **(B)** median filtered skNA signal without QRS interference **(C)** rectified and integrated skNA (iskNA) **(D)** mean baseline value of non-bursting events (pink dotted horizontal line) and burst activity during sympathetic activation event (vertical dashed line) indicated by red dots.

The data from the sensors was processed using filters to remove artifacts such as motion and other high-frequency noise. The ECG signal was processed using a 60 Hz notch filter to remove power line interference, and a seventh order Butterworth band-pass filter between 0.01 and 30 Hz to remove movement artifacts and other high frequency noise (Figure 2). Smoothing is often useful to suppress noise or interference on a signal and was done by using a moving average filter on the signal (Gacek and Pedrycz, 2014), skNA is derived from the ECG signal using a band-pass filter between 500 and 1,000 Hz (Lenis et al., 2017). The skNA signal contained interferences from QRS intervals (Figure 3A). These QRS intervals were isolated through the Pan-Tompkins algorithm and smoothed using a median filter to remove the interference (Figure 3B). The signal was then rectified and integrated (iskNA) over a 100 ms window (Figure 3C). Non-bursting baseline values of iskNA during rest were used to determine bursts in nerve activity. The mean of non-bursting iskNA plus 3 standard deviations (SD) were used as a threshold amplitude for determining bursting activity (Figure 3D).

#### Feature Extraction

**FIGURE 4:**
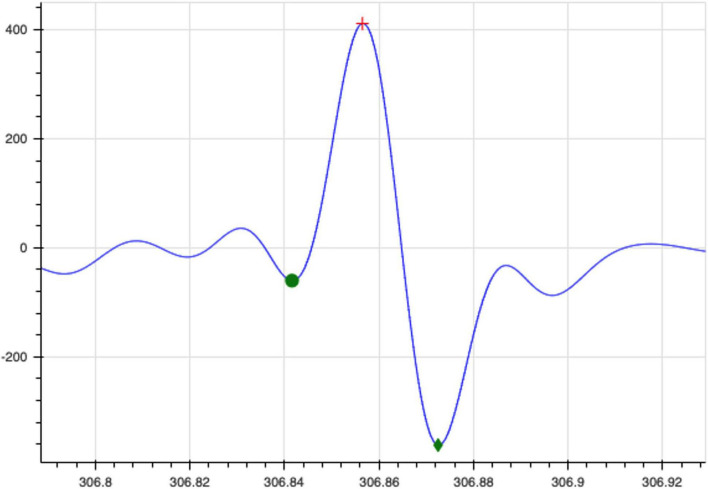
QRS segments identified from each individual beat of the filtered ECG signal.

A fixed, sliding, non-overlapping window of 15 s was used to extract thirty-six relevant features (Table 1). The detection of the QRS complexes and the R-peaks provide the fundamentals for almost all automated ECG analytics (Sadhukhan and Mitra, 2012). The Pan-Tompkins algorithm was used to extract the RR peaks as well as the QRS segments of each beat of the filtered ECG signal (Figure 4). To ensure detection accuracy, the derived RR peaks are further processed to ensure the minimum difference between two successive peaks is between 100 and 500 ms (200 bpm < HR < 600 bpm) to generate the normal to normal (NN) intervals (Chan et al., 2005). The heart rate and medianNN are calculated from the NN intervals. The PR interval, QRS interval, QT interval, ST interval, PR segment and the ST segment which provide additional information about the cardiac condition were also extracted (Schamroth, 1990).

**TABLE 1:** 36 features extracted from the different sensors.

Signal	Features	
ECG	**Temporal**: 1. NN intervals 2. Heart rate 3. QRS interval 4. PR interval 5. **MedianNN** 6. Number of NN intervals < 5 ms (nn5) 7. **Percentage of nn5 (pnn5)** 8. covNN 9. **RMSSD**	**Spectral**: 10. Power of low frequency band (0.01–0.75Hz) - LF_pow_ 11. Power of high frequency band (0.75–2.5 Hz) - HF_pow_ 12. LF_pow_/HF_pow_ 13. Area under low frequency bands (ALF) 14. Area under high frequency bands (AHF) 15. ALF/AHF ratio
skNA	**Temporal:** 16. Average skNA 17. **Average iskNA** 18. Area under Curve (AUC) skNA 19. **Number of bursts** 20. Duration of bursts 21. AUC bursts	**Spectral:** 22. Power of low frequency band (0–2.5 Hz) - LF_*pow*_ 23. Power of high frequency band (2.5–5 Hz) - HF_*pow*_ 24. Power of very high frequency band (5–10 Hz) - VHF_pow_ 25. LF_pow_/HF_pow_ 26. Area under LF band (ALF) 27. Area under HF band (AHF) 28. Area under VHF band (AVHF) 29. ALF/AHF ratio
Skin Temperature	30. Δtemperature 31. Mean temperature 32. Median temperature	
Blood Pressure	33. ΔSBP 34. ΔDBP 35. Mean SBP 36. Mean DBP 37. Mean arterial pressure (MAP)	

*Top 5 selected features have been indicated in bold.*

Heart rate variability (HRV) measures were also calculated from each window. These include the standard deviation of NN beat intervals (SDNN), covariance of NN intervals (covNN), the square root of the mean of the squares of the successive differences between adjacent NNs (RMSSD), and the proportion of the number of successive NN intervals which differ by more than 5 ms (NN_5_) as well as the percentage of NN_5_ (pNN_5_). The spectral power for HRV was analyzed on the windowed ECG segments. The total power (TP), very-low-frequency (VLF; 0.003–0.04 Hz), low-frequency (LF; 0.04–0.15 Hz), high-frequency (HF; 0.15–0.4 Hz) components were extracted from an FFT performed on the ECG signal. The peak amplitudes in VLF, LF, and HF components as well as the areas under these components were calculated. Additionally, the LF/HF ratio was also calculated.

The number of bursts, duration of bursts, Area under curve of the bursts were extracted from the iskNA. In addition, the average value of skNA and iskNA were extracted from each window. In addition, FFT performed on the skNA signal allowed extraction of the low, high and very high frequency bands of the sympathetic nerve activity.

A total of 2,200 data points were collected from the rats. After the features were extracted, they were normalized using a min-max scaler. For each feature value, we computed the *z*-score, that is the number of standard deviations the value was from its mean. Observations with a *z*-score greater than 3 (<8% of the dataset) were considered outliers and removed. Majority of these outliers were non-AD data. Observations containing missing values, though rare, were discarded.

### Feature Selection

For classification and regression tasks, it is often useful to remove features which do not help model accuracy. The removal of extraneous variables tends to lower variance in the predicted values and reduces the likelihood of overfitting. Moreover, determining which features are useful in prediction can help point toward underlying mechanisms of the given problem, from which domain experts can work to develop new hypotheses. Below, we discuss the approaches we used for selecting useful features.

#### Univariate Filter Methods

Univariate feature selection allows the examination of each feature individually to measure its ability to determine the response variable. This often involves the computation of measures of association.

We computed a p-value through hypothesis testing (Student’s *t*-test) and removed any features which did not meet a specific threshold (*p* < 0.05). A chi-squared test was used to determine which features most closely resulted in changes in the features of the predictor.

**FIGURE 5:**
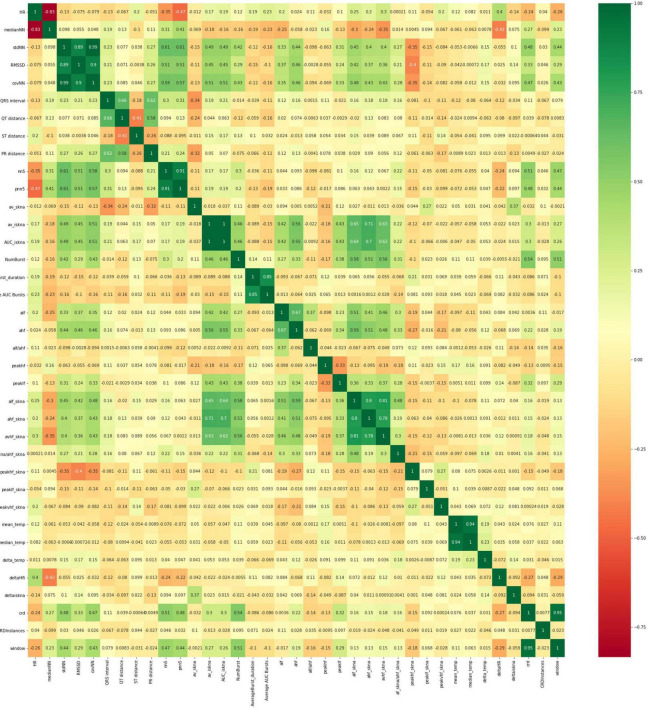
Heatmap of correlation of the thirty-six different features (x and y axes are the features listed in Table 1 above). Highly correlated features are removed and not considered in the development of the models.

We also used Pearson correlation-based feature selection wherein highly correlated features were removed. We removed predictors which are highly correlated (*R*^2^ > 0.7) with other predictors (Figure 5). While this approach is simple and can be reasonably effective, features which show higher-order or multivariate relationships with the response variable (but which individually do not show strong patterns) may unwittingly be discarded.

#### Best Subset Selection and Stepwise Search

Commonly used best subset regression techniques involve fitting and comparing 2*^p^* possible models, wherein p is the number of features. However, this technique is often impractical for all but the smallest number of total features. In our case, with 30 features, 1 billion potential models need to be fit to determine the ones which lead to the best performance metrics. We used an iterative, stepwise, “greedy” search approach wherein a full model is initially built, and features are either successively added or removed from the dataset. We performed “recursive feature elimination” starts by fitting a full model (containing all available features), and computes “feature importance” values for each feature (e.g., for logistic regression, one could use the p-value from the Wald-tests for the coefficient parameters). We also used the inherent abilities of the decision tree to calculate a feature importance score from the Gini coefficient. Features whose feature importance does not meet a specified threshold were discarded. The procedure was then repeated, recursively, until all remaining features meet the threshold criteria, or until a target model dimension is achieved.

#### Recursive Feature Elimination

A recursive feature elimination (RFE) algorithm was used for feature selection. The RFE algorithm method attempts to find the best subset of size σ (σ < N) through a greedy backward selection. It chooses the σ features which lead to the largest margin of class separation by the logistic regression classifier. It iterates in a greedy fashion through the removal of input dimensions/features to decrease the margin of separation between the classes until only σ input dimensions remain. A binary logistic regression model was used for classification to identify the impact of the different features in predicting the onset of AD.

### Machine Learning Models

Eleven different classifiers were compared for the initial exploration of performance. These include K- Nearest Neighbor (KNN), linear and logistic regression, support vector machines (SVM) with linear and RBF kernels, Naïve Bayes, Quadratic Discriminant Analysis, ensemble methods such as random forest and Adaboost models, and neural networks (multilayer perceptron).

In order to train our machine learning models, we split the data into three stratified sets- the training set (70%), the test set (15%) and the validation set (15%). 10-fold cross-validation (CV) was used to create variations of the training, test and validation sets to reduce overfitting. The models were trained on the complete dataset as well as the reduced dataset developed from the feature selection methods.

### Performance Measures

We measured performance through a confusion matrix (Table 2). To determine the best performing algorithm, we used metrics of accuracy, sensitivity (true positive rate), specificity (true negative rate), and AUC-ROC score to evaluate the performance of the different models developed using the different feature selection techniques. Through the ROC curves, we were able to screen for the different types of errors which arise in many biomedical scenarios.

**TABLE 2:** Representation of the confusion matrix for AD detection and metrics determination.

	Predicted AD	Predicted Non-AD
Actual AD	True positive (TP)	False negative (FN)
Actual non-AD	False positive (FP)	True negative (TN)

*Sensitivity: true positive rate; specificity: true negative rate.*

## Results

Through the aforementioned feature selection approaches, we identified five relevant features which best characterized the onset of AD. These five features include medianNN, average iskNA, number of bursts, which are representative of sympathetic activity and RMSSD, pNN5 which are representative of vagal activity. These five features enabled a deeper insight into the biological processes involved in the resulting symptoms of AD (Suresh et al., under review).

**FIGURE 6:**
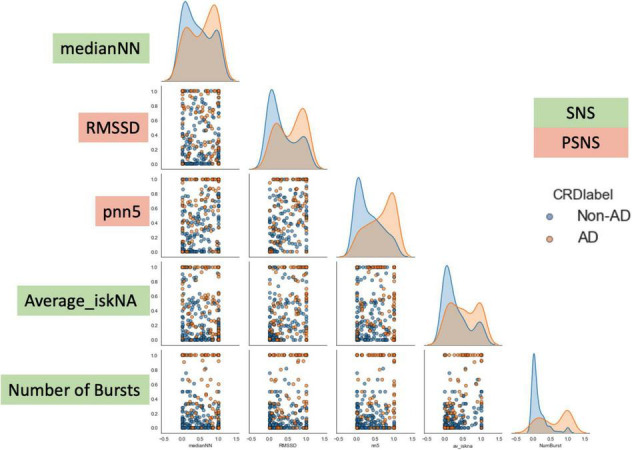
Bivariate plot showing the differences observed in the five features during AD and non-AD events. There is an observed overlap between the two classes but also some differences between the features which make them discernible. The y-axis are the normalized units of each feature. The green boxes are features which represent sympathetic activity while the red boxes are feature which represent vagal activity.

As can be observed from Figure 6, there is an observed overlap when visualizing AD and non-AD responses on a bivariate plot. However, the differences in the different distributions suggest the ability for discernment between the presence and absence of AD through the five features. These formed the basis of the separation between the two classes (AD and non-AD).

The reduced subset of features enabled us to develop and compare the eleven different models (Table 3). The best performing machine learning model developed using the reduced feature subset was a five-layer neural network (multi-layer perceptron) which had high accuracy (93.4%), sensitivity (93.5%) and specificity (93.3%). There is a notable increase in performance of the neural network when trained on the reduced feature subset when compared to the dataset without any feature selection.

**TABLE 3:** Performance metrics for the different classifiers with the AD dataset.

Name	Accuracy (%)	Sensitivity (%)	Specificity (%)	AUC-ROC
Neural network (without feature selection)	72.2	70.1	76.7	0.74
**Neural network**	**93.4**	**93.5**	**93.3**	**0.93**
Adaboost	79.3	79.3	79.2	0.78
Decision tree	86.1	83.3	89.5	0.86
Gaussian process	91.7	88.9	94.4	0.92
K Nearest neighbor	86.5	83.3	89.5	0.86
Linear SVM	62.2	30.1	86.7	0.61
Logistic regression	87.4	84.3	82.5	0.87
RBF SVM	63.9	72.2	84.2	0.64
Naïve bayes	88.9	94.4	83.3	0.89
Random forest	63.9	72.2	84.2	0.64

## Discussion

Feature selection performs a reduction in the complexity of a dataset to enable the development of reliable machine learning models (Saeys et al., 2007). Through better feature selection, it is possible to develop models which use physiological and healthcare data as an invaluable data source to assist in disease detection, rehabilitation and treatment (Faust and Bairy, 2012). In this paper, we compared different feature selection methods and machine learning models which enabled us to characterize the onset of AD with high-performance metrics.

These techniques can be used in different capacities to enable the development of machine learning models which are explainable, relevant and most importantly, perform well with clinically relevant physiological data. Machine learning models can enable early mitigation of AD leading to a reduction in related complications and mortality in individuals with SCI.

### Relevance of Feature Selection Using Small Physiological Datasets

With an increase in availability of wearable sensing technologies, such as the Apple™ Watch, Fitbit™, there is an increasing amount of healthcare data that can be collected and made available to clinicians and others in the field of healthcare. This leads to a voluminous number of features, which can be extracted allowing a richer understanding of the biological processes involved in various disease states instead of being limited in collecting data in controlled settings. Unfortunately, this development of increasingly complex datasets which have a great deal of inter-related features serves to complicate straightforward discrimination of results necessitating the development of machine learning models. There is a need to provide efficient, parallel data processing techniques to develop efficient machine learning models, which is made possible through feature selection (Jain and Singh, 2018). Feature selection is particularly important when making predictions regarding the outcomes or onset of diseases.

Through the feature selection approaches presented in this paper, we were able to narrow our feature subset. The selection of five features rather than thirty-six enabled a sharper focus on relevant changes occurring in the physiology due to the onset of AD. However, there is no “best” feature selection procedure, as the choice of selection procedure highly depends on details of the problem at hand: the number of features, the availability of feature importances, and the computational resources required by the model fitting procedure. The techniques presented in this paper provide a template which can be modified to suit the needs of other small dataset related projects.

### Relevance of Neural Network Performance

From our experiments, the feedforward neural network arguably showed the strongest overall performance, including the highest accuracy and AUC score among the models tested. The Gaussian Process model performed similarly, but with slightly lower accuracy and AUC score. These results indicate that there are likely important non-linear relationships within our data, as neural networks and Gaussian processes are two of the more flexible supervised learning models. In our case, the neural network contained a total of ∼2,000 parameters (and Gaussian processes are non-parametric). It is not too surprising that these two models performed similarly, as it is known that neural networks, in a sense, approximate Gaussian processes (Quiñonero-Candela and Rasmussen, 2005).

A drawback of the more flexible models is that they tend to require relatively more data to achieve good performance. On the other hand, as the size of data grows, they tend to better detect subtle relationships that may exist. Consequently, as more data becomes available, we may likely see even further improvements in the performance of the neural network and Gaussian process models (as well as the other more flexible models).

We do note that although the more flexible models showed the strongest performance, two of the simpler models—logistic regression and quadratic discriminant analysis—showed reasonably strong performance as well. This suggests that while complex non-linear relationships may exist within the data, much of the variation in the response is accounted for by first and second-order terms of the features. In a setting where the number of observations is relatively small, it may be more prudent to consider the simpler methods, as they tend to be relatively more stable (low variance), especially for smaller datasets.

### Clinical Relevance in Autonomic Dysreflexia

Recognition and prevention of AD related signs and symptoms plays a critical role in avoiding escalation to more dire circumstances in clinical and non-clinical environments. Currently the standard approach for managing AD is to train persons with SCI to recognize their symptoms and to promptly alleviate the AD trigger, which can be difficult to identify and frequently requires the assistance of a caregiver. There is a need for a sensitive yet non-invasive method of detecting the onset of AD, which can be adopted easily into clinical practice and for at home use (Hubli and Krassioukov, 2014).

The major findings of this study suggest that there are alternate techniques to determining the onset of AD through non-invasive wearable sensing techniques. Additionally, there are signatures of the onset of AD described through these five relevant features which could enable better detection (Suresh et al., unpublished).

These could be complementary to current clinical tools. A non-invasive sensor system that can automatically detect the onset of AD, can improve independence and quality of life of individuals with SCI. Additionally, such a detection system could allow individuals more time to identify and eliminate the trigger before escalation to dangerous hypertensive levels.

## Data Availability Statement

The raw data supporting the conclusions of this article will be made available by the authors, without undue reservation.

## Ethics Statement

The animal study was reviewed and approved by the Purdue University Institutional Animal Care and Use Committee.

## Author Contributions

SS performed all data collection and analysis of results with assistance from DN. TE provided expertise in skin nerve activity procedures and analyses. GL assisted with data science techniques and methodologies. BD worked with SS on experimental design and interpretation of results as well as study oversight. All authors contributed to the article and approved the submitted version.

## Conflict of Interest

The authors declare that the research was conducted in the absence of any commercial or financial relationships that could be construed as a potential conflict of interest.

## Publisher’s Note

All claims expressed in this article are solely those of the authors and do not necessarily represent those of their affiliated organizations, or those of the publisher, the editors and the reviewers. Any product that may be evaluated in this article, or claim that may be made by its manufacturer, is not guaranteed or endorsed by the publisher.

## References

[B1] Alfaras M. Soriano M. C. Ortín S. (2019). A fast machine learning model for ECG-based heartbeat classification and arrhythmia detection. *Front. Phys.* 7:103. 10.3389/fphy.2019.00109

[B2] Alimadadi A. Aryal S. Manandhar I. Munroe P. B. Joe B. Cheng X. (2020). Artificial intelligence and machine learning to fight COVID-19. *Physiol. Gen.* 52 200–202. 10.1152/physiolgenomics.00029.2020 32216577 PMC7191426

[B3] Badillo S. Banfai B. Birzele F. Davydov I. I. Hutchinson L. Kam-Thong T. (2020). An introduction to machine learning. *Clin. Pharmacol. Therapeut.* 107 871–885. 10.1002/cpt.1796 32128792 PMC7189875

[B4] Chan H. L. Chou W. S. Chen S. W. Fang S. C. Liou C. S. Hwang Y. S. (2005). Continuous and online analysis of heart rate variability. *J. Med. Eng. Technol.* 29 227–234. 10.1080/03091900512331332587 16126583

[B5] Chen M. Hao Y. Hwang K. Wang L. Wang L. (2017). Disease prediction by machine learning over big data from healthcare communities. *IEEE Access* 5 8869–8879. 10.1109/ACCESS.2017.2694446

[B6] Daugherty A. Rateri D. Hong L. Balakrishnan A. (2009). Measuring blood pressure in mice using volume pressure recording, a tail-cuff method. *J. Vis. Exp.* 15:1291. 10.3791/1291 19488026 PMC2794298

[B7] Esteva A. Kuprel B. Novoa R. A. Ko J. Swetter S. M. Blau H. M. (2017). Dermatologist-level classification of skin cancer with deep neural networks. *nature* 542 115–118. 28117445 10.1038/nature21056 PMC8382232

[B8] Everett T. H. Doytchinova A. Cha Y. M. Chen P. S. (2017). Recording sympathetic nerve activity from the skin. *Trends. Card. Med.* 27 463–472. 10.1016/j.tcm.2017.05.003 28619579 PMC5753603

[B9] Faust O. Bairy M. G. (2012). Nonlinear analysis of physiological signals: a review. *J. Mechan. Med. Biol.* 12:1240015.

[B10] Gacek A. Pedrycz W. (2014). *ECG signal Processing, Classification and Interpretation: A Comprehensive Framework of Computational Intelligence.* Berlin: Springer Scienc.

[B11] Hubli M. Krassioukov A. V. (2014). Ambulatory blood pressure monitoring in spinal cord injury: clinical practicability. *J. Neur.* 31 789–797. 10.1089/neu.2013.3148 24175653 PMC3997095

[B12] Jain D. Singh V. (2018). Feature selection and classification systems for chronic disease prediction: a review. *Egyptian Inform. J.* 19 179–189.

[B13] Jen C.-H. Wang C.-C. Jiang B. C. Chu Y.-H. Chen M.-S. (2012). Application of classification techniques on development an early-warning system for chronic illnesses. *Exp. Syst. Appl.* 39 8852–8858.

[B14] Jiang Z. Zhao Y. Doytchinova A. Kamp N. J. Tsai W. C. Yuan Y. (2015). Using skin sympathetic nerve activity to estimate stellate ganglion nerve activity in dogs. *Heart Rhythm* 12 1324–1332. 10.1016/j.hrthm.2015.02.012 25681792 PMC4442039

[B15] Jothi N. Husain W. (2015). Data mining in healthcare–a review. *Procedia comput. Sci.* 72 306–313.

[B16] Kuhn M. Johnson K. (2013). *Applied Predictive Modeling.* Berlin: Springer.

[B17] Lenis G. Pilia N. Loewe A. Schulze W. H. W. Dössel O. (2017). Comparison of baseline wander removal techniques considering the preservation of st changes in the ischemic ECG: A Simulation Study. *Comput. Mathemat. Method. Med.* 2017 1–13. 10.1155/2017/9295029 28373893 PMC5361052

[B18] LI Gang Z. Y. (2005). Principle and Application of 1Wire Bus Digital Thermometer DS18B20. Modern Electronic Techniques, 21.

[B19] Maxim (2008). *Maxim DS18B20 thermometer datasheet. 22.* Available online at: https://cdn-shop.adafruit.com/datasheets/DS18B20.pdf (accessed September 9, 2020).

[B20] Mishra T. Wang M. Metwally A. A. Bogu G. K. Brooks A. W. Bahmani A. (2020). Early Detection Of COVID-19 using a smartwatch. *medRxiv* [Preprint]. 10.1101/2020.07.06.20147512 PMC9020268 33208926

[B21] O’ Mahony S. M. Tramullas M. Fitzgerald P. Cryan J. F. (2012). Rodent models of colorectal distension. *Current Protocol. Neurosci.* 61 1–13. 10.1002/0471142301.ns0940s61 23093353

[B22] Polat K. Gunes S. (2007). Detection of ECG Arrhythmia using a differential expert system approach based on principal component analysis and least square support vector machine. *Appl. Mathemat. Comput.* 186 898–906. 10.1016/j.amc.2006.08.020

[B23] Popok D. West C. Frias B. Krassioukov A. V. (2016). Development of an algorithm to perform a comprehensive study of autonomic dysreflexia in animals with high spinal cord injury using a telemetry device. *J. Visual. Exp.* 113, e52809–e52809. 10.3791/52809 27500446 PMC5091701

[B24] Quiñonero-Candela J. Rasmussen C. E. (2005). A unifying view of sparse approximate gaussian process regression. *J. Mach. Learn. Res.* 6 1939–1959.

[B25] Rabchevsky A. G. Patel S. P. Lyttle T. S. Eldahan K. C. O’Dell C. R. Zhang Y. (2012). Effects of gabapentin on muscle spasticity and both induced as well as spontaneous autonomic dysreflexia after complete spinal cord injury. *Front. Physiol.* 3:329. 10.3389/fphys.2012.00329 22934077 PMC3429097

[B26] Radha M. De Groot K. Rajani N. Wong C. C. P. Kobold N. Vos V. (2019). Estimating blood pressure trends and the nocturnal dip from photoplethysmography. *Physiol. Measur.* 40:025006. 10.1088/1361-6579/ab030e 30699397

[B27] Roski J. Bo-Linn G. W. Andrews T. A. (2014). Creating value in health care through big data: opportunities and policy implications. *Health Affair.* 33 1115–1122. 10.1377/hlthaff.2014.0147 25006136

[B28] Rumshisky A. Ghassemi M. Naumann T. Szolovits P. Castro V. M. McCoy T. H. (2016). Predicting early psychiatric readmission with natural language processing of narrative discharge summaries. *Translat. psychiatry* 6:e921–e921. 10.1038/tp.2015.182 27754482 PMC5315537

[B29] Sadhukhan D. Mitra M. (2012). R-Peak detection algorithm for ecg using double difference and rr interval processing. *Procedia Technol.* 4 873–877. 10.1016/j.protcy.2012.05.143

[B30] Saeys Y. Inza I. Larrañaga P. (2007). A review of feature selection techniques in bioinformatics. *Bioinformatics* 23:2507–2517. 10.1093/bioinformatics/btm344 17720704

[B31] Saria S. Rajani A. K. Gould J. Koller D. Penn A. A. (2010). Integration of early physiological responses predicts later illness severity in preterm infants. *Sci. Transl. Med.* 2:48ra65. 10.1126/scitranslmed.3001304 20826840 PMC3564961

[B32] Schamroth L. (1990). *An Introduction to Electrocardiography 7th ed.* Oxford: University of Oxford.

[B33] Su C.-T. Wang P.-C. Chen Y.-C. Chen L.-F. (2012). Data mining techniques for assisting the diagnosis of pressure ulcer development in surgical patients. *J. Med. Systems* 36 2387–2399. 10.1007/s10916-011-9706-1 21503743

[B34] Suresh S. Duerstock B. S. (2020). *Detection of Dysautonomia in Spinal Cord Injury Through Non-invasive Multi-modal Sensing and Machine Learning.* West Lafayette, IN: Purdue University.

[B35] Suresh S. Everett T. H. Li J. Walls E. K. Duerstock B. (2019). *Sensing Sympathetic Activation Using Novel Non-Invasive Techniques in Rats. in 2019 IEEE Sensors, ed.* Piscataway, NJ: IEEE.

[B36] Uçar M. K. Bozkurt M. R. Bilgin C. Polat K. (2017). Automatic detection of respiratory arrests in OSA patients using PPG and machine learning techniques. *Neural Comput. Appl.* 28 2931–2945. 10.1007/s00521-016-2617-9

[B37] Waring J. Lindvall C. Umeton R. (2020). Automated machine learning: review of the state-of-the-art and opportunities for healthcare. *Artificial Intell. Med* 104:101822. 10.1016/j.artmed.2020.101822 32499001

[B38] Wiens J. Shenoy E. S. (2018). Machine learning for healthcare: on the verge of a major shift in healthcare epidemiology. *Clin. Infect. Diseases* 66 149–153. 10.1093/cid/cix731 29020316 PMC5850539

[B39] Wosiak A. Kowalski R. (2020). Automated feature selection for obstructive sleep apnea syndrome diagnosis. *Procedia Comput. Sci.* 176 1430–1439.

